# Supplementary material for: A CRISPR/Cas9-Based Assay for High-Throughput Studies of Cancer-Induced Innervation
Source: Cancers (Basel). 2023 Mar 29;15(7):2026. doi: 10.3390/cancers15072026 (PMC10093009; doi:10.3390/cancers15072026)
Supplement: Supplementary file 1 [file cancers-15-02026-s001.zip › cancers-2269651-supplementary/cancers-2269651-Figure S1.pdf]

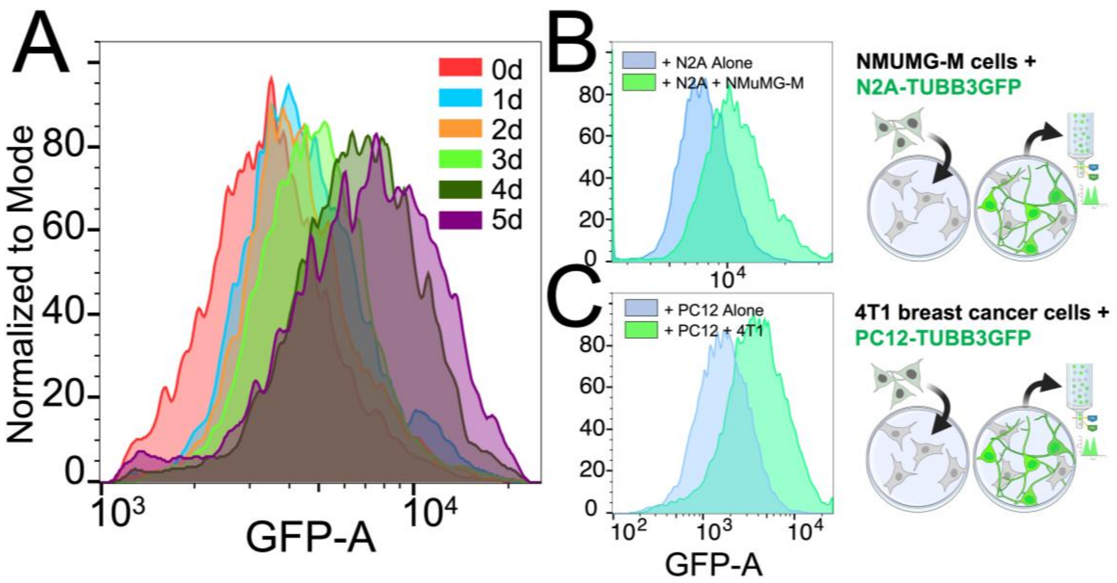

Figure S1. Neuro2A and PC12 models of neuronal differentiation. (A) Flow cytometry analysis of the time response of the neuronal differentiation process in the Neuro2A cell model (N2A-TUBB3-GFP) stimulated with NGF (100 ng/mL) for 0 to 5 days. (B) N2A-TUBB3-GFP cells were cultivated as unstimulated monoculture (Alone) and incubated in direct coculture with NMuMG-M cells (+NMuMG). (C) PC12-TUBB3-GFP cells were cultivated as unstimulated monoculture (Alone) and incubated in direct coculture with 4T1 breast cancer cells (+4T1).
