# Supplementary figures and images for: A CRISPR/Cas9-Based Assay for High-Throughput Studies of Cancer-Induced Innervation
Source: Cancers (Basel). 2023 Mar 29;15(7):2026. doi: 10.3390/cancers15072026 (PMC10093009; doi:10.3390/cancers15072026)

FIGURE 3

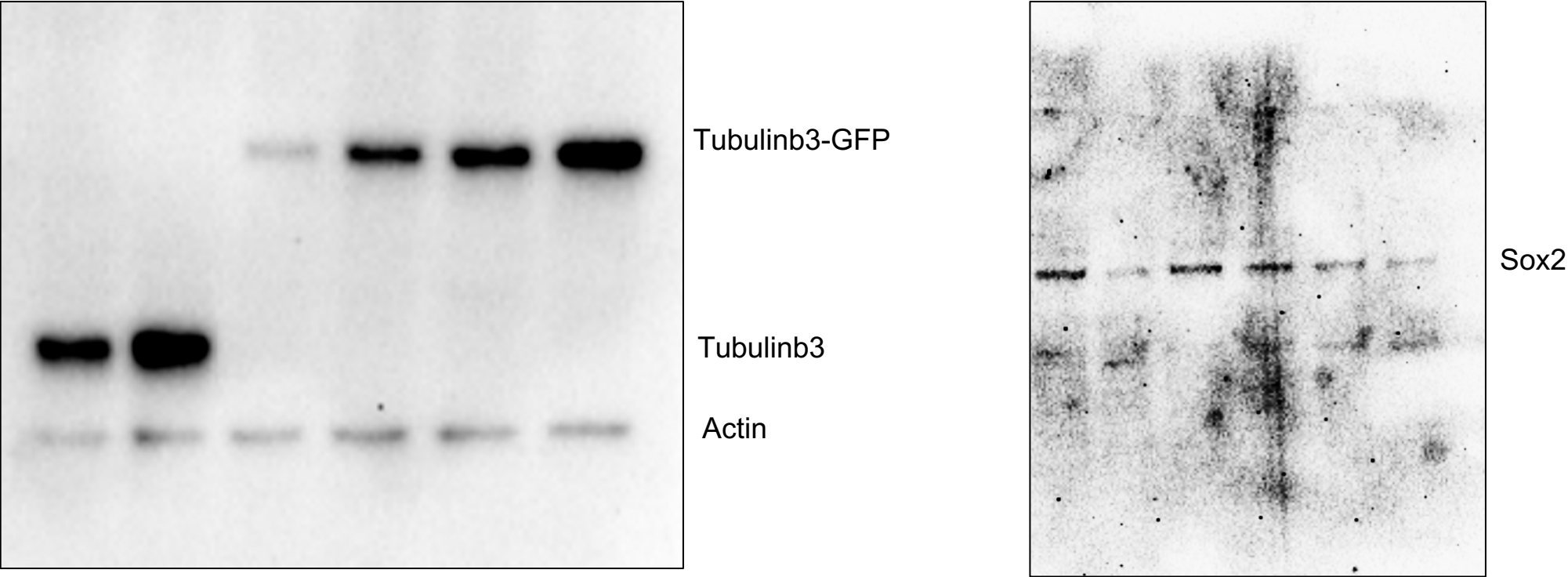

FIGURE 5

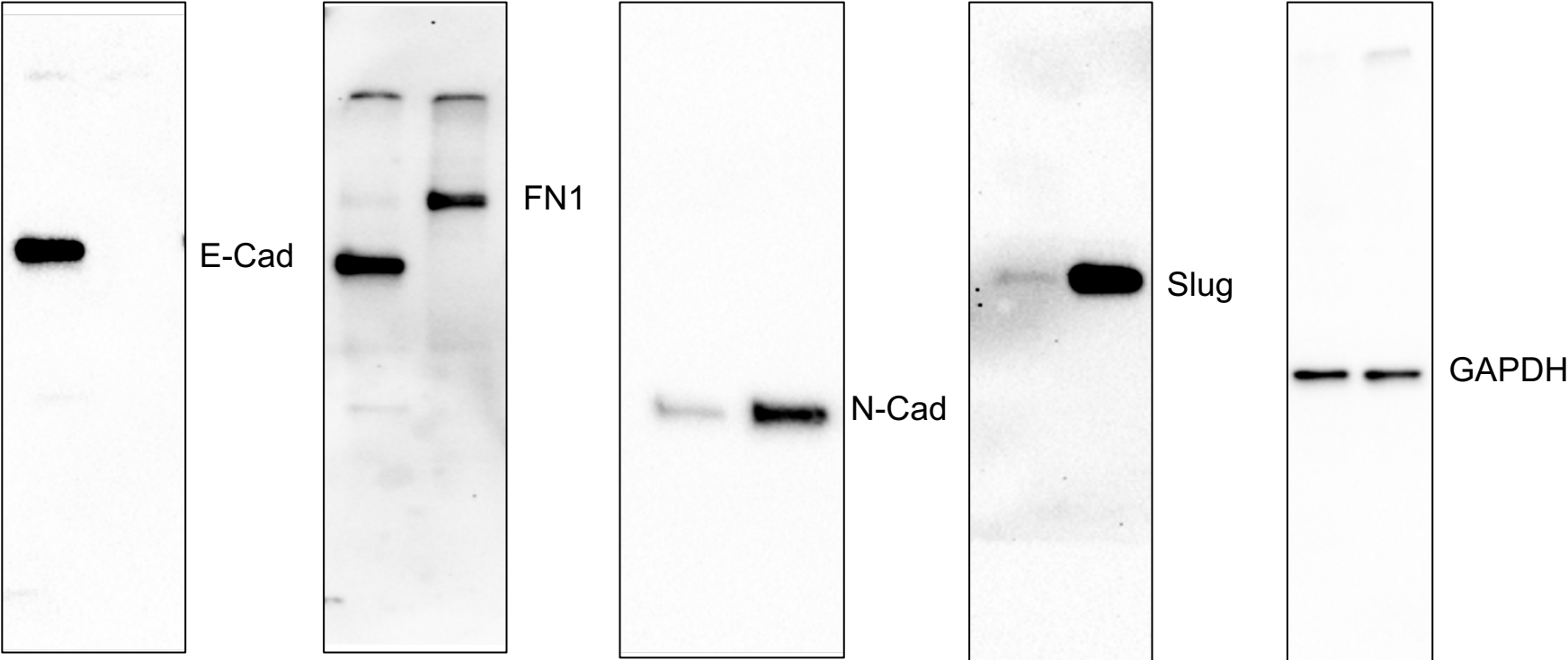

Supplement: Supplementary file 1 [file cancers-15-02026-s001.zip › cancers-2269651-supplementary/cancers-2269651-Supplementary File S1.pdf]
